# Systematic review and meta-analysis of the clinical features of MGRS

**DOI:** 10.1186/s12882-024-03458-5

**Published:** 2024-01-16

**Authors:** Jingxue Du, Zhangxue Hu

**Affiliations:** Department of Nephrology, West China Hospital, Sichuan University, Guoxue Alley, 37#, Wuhou District, 610041 Chengdu, Sichuan Province China

**Keywords:** Monoclonal gammopathy, Renal damage, Clinicopathological characteristics, Meta-analysis

## Abstract

**Background:**

It is crucial to identify patients with monoclonal gammopathy of renal significance (MGRS) from those without MGRS but with monoclonal gammopathy and concomitant kidney diseases. However, there have been few studies with large sample sizes, and their findings were inconsistent. This study aimed to conduct a meta-analysis of MGRS to describe the general characteristics of MGRS and its predictive factors.

**Methods:**

Cohort or case-control studies published through December 2022 and related to clinicopathological features of MGRS were retrieved from the PubMed, Cochrane Library, Web of Science, Scopus, and Embase databases. Two researchers searched for studies that met the inclusion criteria. In the univariate analysis, fixed- or random- effects models were used to obtain pooled estimates of the weighted mean difference (WMD) and odds ratio (OR) for risk factors. In the multivariate analysis, the ORs of the independent risk factors from each study were pooled after transforming the original estimates.

**Results:**

The meta-analysis included six studies. Univariate analysis showed that the following variables were statistically significant in MGRS: age (WMD = 1.78, 95%CI 0.21–3.35), hypertension (OR = 0.54, 95%CI 0.4–0.73), diabetes (OR = 0.42, 95%CI 0.29–0.59), albumin (WMD = − 0.26, 95%CI − 0.38–−0.14), urinary protein level (WMD = 0.76, 95%CI 0.31–1.2), urinary protein ≥ 1.5 g/d (OR = 1.98, 95%CI 1.46–2.68), lambda-chain value (WMD = 29.02, 95%CI 16.55–41.49), abnormal free light-chain ratio (OR = 4.16, 95%CI 1.65–10.47), bone marrow puncture rate (OR = 5.11, 95% CI 1.31–19.95), and abnormal bone marrow outcome rate (OR = 9.63, 95%CI 1.98–46.88). Multivariate analysis showed urinary protein ≥ 1.5 g/d (OR = 2.80, 95%CI 1.53–5.15) and an abnormal free light-chain ratio (OR = 6.98, 95%CI 4.10–11.91) were associated with predictors of MGRS.

**Conclusions:**

Compared with non-MGRS patients with monoclonal gammopathy and concomitant kidney diseases, patients with MGRS were older, had fewer underlying diseases, more urinary protein, more abnormal free light-chain ratio, and more abnormal bone marrow results. Urinary protein ≥ 1.5 g/d and an abnormal free light-chain ratio were independent risk factors for MGRS.

## Introduction

Monoclonal gammopathies (MGs) are a group of plasma cell- or B lymphocyte-proliferative disorders that create monoclonal immunoglobulin (MIg). MGs include malignant neoplasms (multiple myeloma, Waldenstrom’s macroglobulinemia, lymphoma), and nonmalignant minor clonal proliferative disorder, termed monoclonal gammopathy of undetermined significance (MGUS). MIg can harm different organs via tumor burden-related mechanisms (hyperviscosity, cast nephropathy) and non-tumor burden pathways (varying types of deposition in tissues). The diverse organ injuries caused by MIg via non-tumor burden pathways have been receiving increasing attention. Monoclonal gammopathy of clinical significance (MGCS) was proposed to identify a MG featuring two main characteristics: a quiescent underlying clone and symptoms that are related to the MIg or the clone itself by mechanisms other than the tumor burden [1]. Even though these conditions do not meet the criteria for diagnosis or treatment of MIg-related tumors, urgent therapy must be started because of organ damage [2]. 

MIg-related renal injury is common in MGCS, and MG of renal significance (MGRS) is used to describe these patients [3]. These disorders can’t meet the criteria for diagnosis or initiation of treatment for MIg-related tumors (such as multiple myeloma with CRAB criteria, symptomatic Waldenstrom’s macroglobulinemia and lymphoma). Most MGRS patients should be treated to suppress the MIg production to alleviate renal injury or prevent recurrence after transplantation [4, 5]. As is commonly understood, patients with MGUS only have a minor clone that is not yet malignant, do not have any end-organ damage, and should not receive anti-tumor treatment [6]. Patients with MGUS may have accompanying MIg-unrelated renal diseases. It is essential to distinguish between MGRS and MGUS.

The spectrum of MGRS is broad and diverse [7]. However, there are few studies concerning the general characteristics of MGRS, and the results are inconsistent and depend on the study type, sample size, geography, and statistical methodologies [8, 9]. The aim of this meta-analysis was to pool published MGRS studies to determine the prevalence of MGRS in patients with MG and renal injury, characterize the spectrum of MGRS, and explore clinical indicators of MGRS.

## Materials and methods

Our meta-analysis was registered with PROSPERO (PROSPERO identifier CRD42023396439) and reported following the guidelines of the Meta-analysis of Observational Studies in Epidemiology statement [10]. 

### Literature search

Articles about MGRS were retrieved from the PubMed, Cochrane Library, Web of Science, Scopus, and Embase databases. To conduct a more thorough search for MGRS-related literature, we used the phrases “MGRS” or “monoclonal gammopathy of renal significance” in our searches (We initially searched the articles by combining the acronym or complete name of MGRS, risk factors or clinical features, and research type, but only a few studies were located). The meta-analysis covered articles published before December 2022. A reference search was also performed.

### Selection of studies

Two independent reviewers evaluated all potential articles. All candidate articles had to meet the following criteria: (1) all literature on MGRS from the establishment of the database to December 2022 for which the research type was a case-control study or cohort study; (2) all patients with MGRS were diagnosed by renal biopsy and met the diagnostic criteria for MGRS proposed by the International Kidney and Monoclonal Gammopathy Research Group (IKMG); [3] (3) the effect size could be directly extracted from the studies or calculated from the data in the articles; and (4) written in English. Any disputes were settled by discussion.

### Data extraction and quality assessment

Two researchers extracted data from the articles independently. We extracted the following information: the first author’s name, publication date, study region, research design type, demographic data, effect sizes for clinical characteristics between the two groups, independent risk variables of MGRS patients and their odds ratio (OR), and 95% confidence intervals. We assessed these studies using the Newcastle–Ottawa Scale (NOS) in which the score ranges from zero to nine points [11]. We preferred trials with ≥ six points available for our meta-analysis. Any issues were settled through discussion.

### Statistical analysis

STATA 17.0 software (STATA, College, TX) was used to perform all analyses. We used *I*^*2*^ statistics to perform all heterogeneity tests, and an *I*^*2*^ > 50% was considered significant. For *I*^*2*^ > 50%, we used the random-effects model for meta-analysis. For *I*^*2*^ ≤ 50%, a fixed-effects model was adopted. In the univariate analysis, the weighted mean difference (WMD) was used to analyze continuous variables; for binary variables, the OR was used to analyze the clinical characteristics of MGRS. For the estimated prevalence and multivariate analysis, the prevalence of MGRS in MG patients with renal pathology and ORs of the independent risk factors obtained from each study were pooled after transforming the original estimates. The level of statistical significance was set to *P* < 0.05. Due to the limited number of included studies, this study did not conduct further subgroup analysis, sensitivity analysis, meta-regression analysis, or publication bias analysis.

## Results

### Study selection and study characteristics

**Fig. 1:**
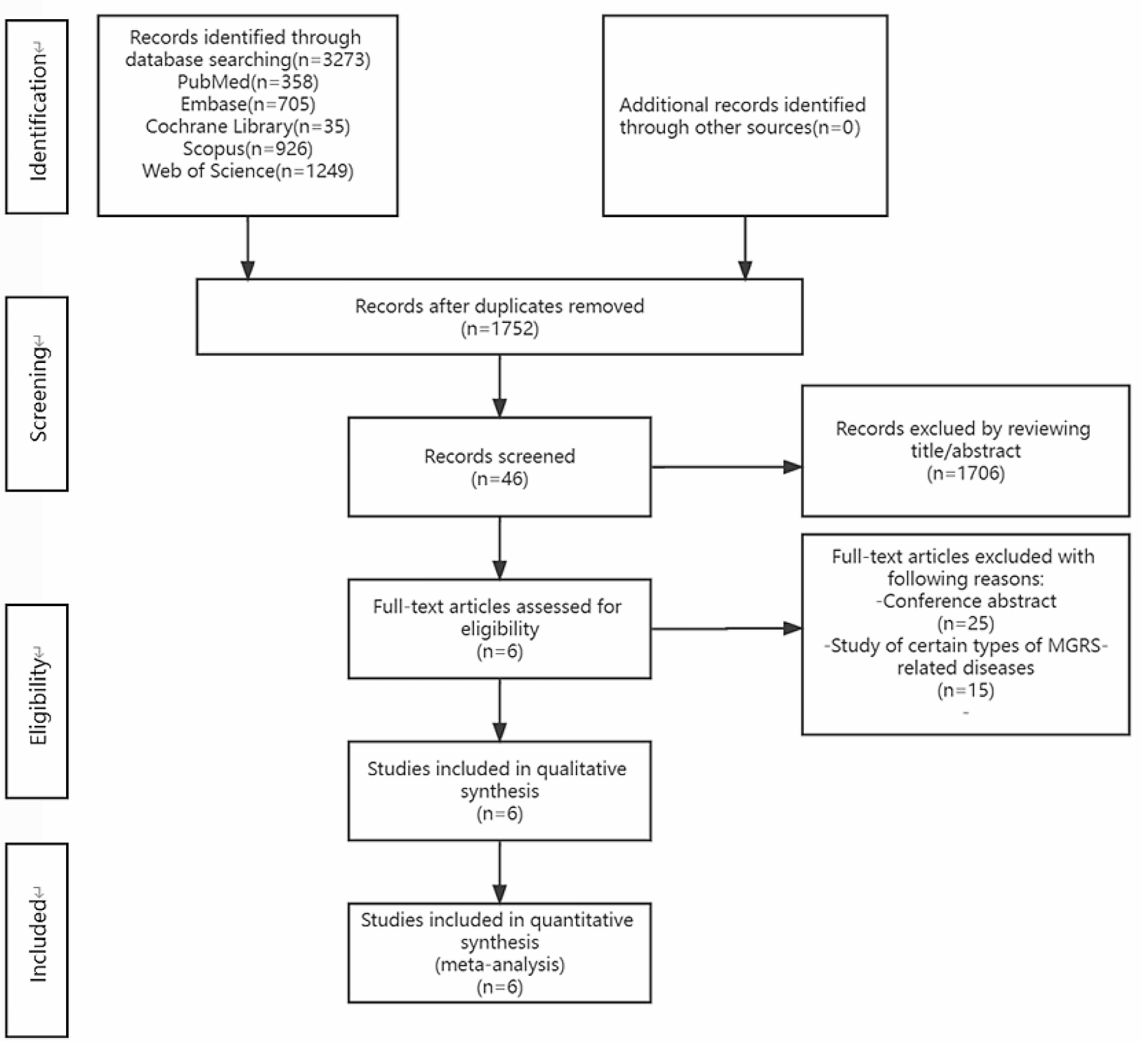
PRISMA flow diagram

A total of 3273 studies were retrieved, but only 46 were evaluated after deleting the duplicate articles. Six studies met the inclusion criteria after further screening and were included in the final meta-analysis [8, 9, 12–15]. The literature screening process is shown in Fig. 1. The main features and NOS results of the included studies are shown in Table 1.

**Table 1:** Baseline characteristics of included studies [8, 9, 12–15]

Author(year)	Country	Study design	Case	Control	Prevalence (MG patients who underwent renal biopsy)	NOS
Yong et al.(2022)	China	Case-control study	261	426	38%	7
Klomjit et al. (2020)	America	Case-control study	64	96	40%	7
Tang et al.(2021)	China	Case-control study	42	19	/	6
Nie et al.(2021)	China	Case-control study	43	42	51%	6
Yu et al.(2020)	China	Case-control study	187	/	/	6
Gozzetti et al.(2022)	Europe and America	Case-control study	280	/	/	7

NOS = Newcastle–Ottawa Scale

### Meta-analysis results

#### Classification spectrum of MGRS-related disorders and prevalence of MGRS in patients with MG who underwent renal biopsy

**Fig. 2:**
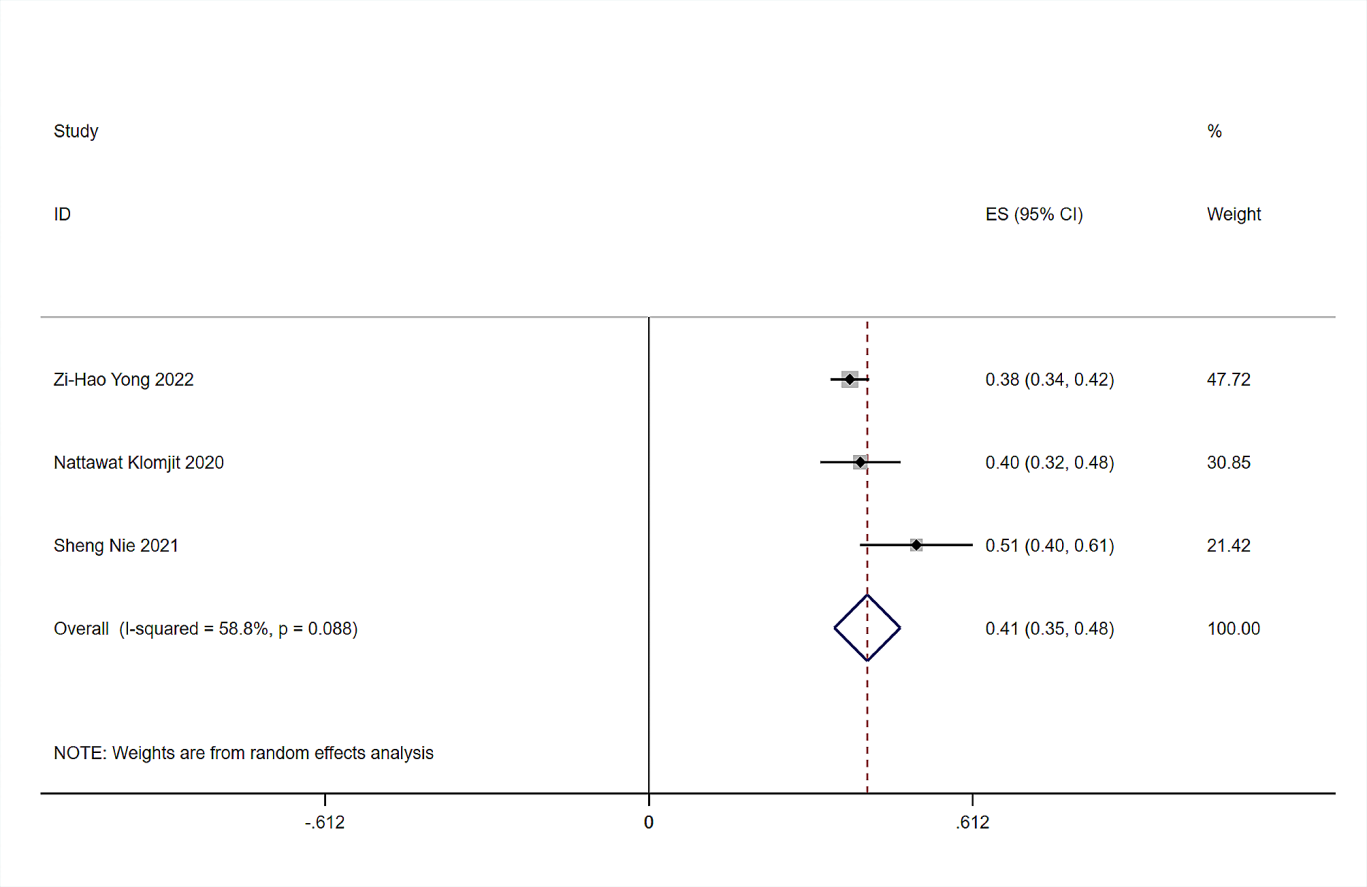
Forest plot for prevalence analysis of MGRS in patients with MG who underwent renal biopsy

**Fig. 3:**
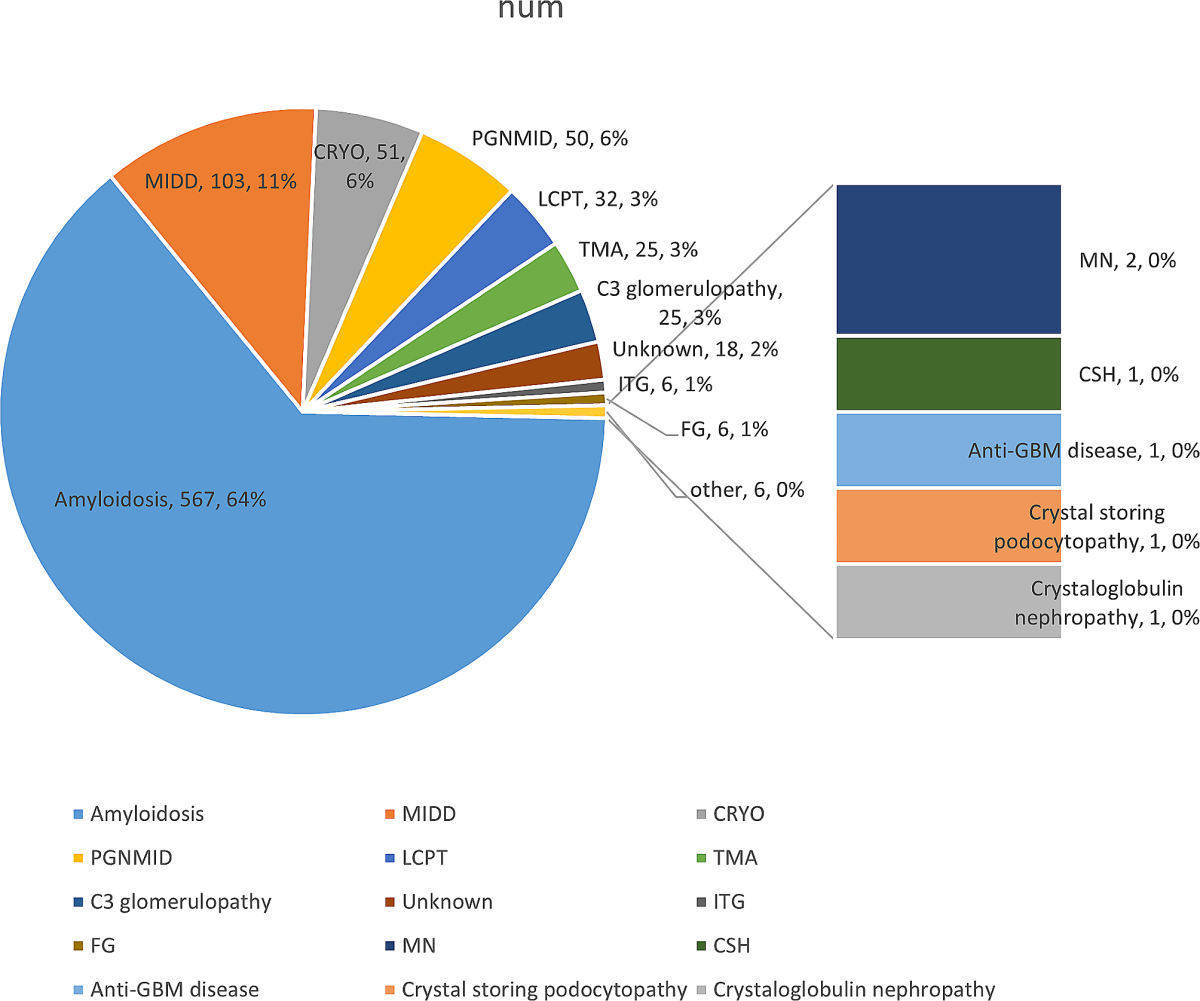
Pie chart for analysis of the percentage of MGRS-related lesions. PGNMID, proliferative glomerulonephritis and monoclonal immunoglobulin deposits. FG, fibrillary glomerulonephritis.MIDD,monoclonal immunoglobulin deposition disease. LCPT, light-chain proximal tubulopathy. CRYO, cryoglobulinaemic glomerulonephritis; TMA, thrombotic microangiopathy. ITG, Immunotactoid glomerulonephritis. CSH, crystal-storing histiocytosis

In our research, 877 individuals with MGRS and 583 controls from six studies were enrolled. The prevalence of MGRS varied from 38 to 51% [8, 9, 13]. The prevalence of MGRS overall was 41% (95% CI 0.35–0.48, *I*^*2*^ = 58.8%, *P* < 0.0001) after performing a meta-analysis using a random-effects model with all of the data. The forest plots are shown in Fig. 2. The spectrum of MGRS-related diseases is shown below (Fig. 3). Amyloidosis was most prevalent, followed by monoclonal immunoglobulin deposition disease (MIDD). Notably, patients whose pathological diagnosis did not meet the classification of MGRS but did show monotypical light-chain deposits in renal tissue were classified as “unknown” [13, 15]. 

#### Meta-analysis of MGRS clinical features

We individually assessed pooled effect sizes for 19 clinical characteristics (age, sex, hypertension, diabetes, albumin, hemoglobin, serum creatinine, estimated glomerular filtration rate, complement C3, complement C4, low complement C3 ratio, urinary protein level, urinary protein ≥ 1.5 g/d, hematuria, kappa-chain value, lambda-chain value, abnormal free light-chain ratio (kappa / lambda chain), bone marrow puncture rate, and abnormal bone marrow outcome rate). The *P* value was statistically significant among the following ten clinical features: age (WMD = 1.78, *P* = 0.026), hypertension (OR = 0.54,, *P* < 0.001), diabetes (OR = 0.42, *P* < 0.001), albumin (WMD = − 0.26, *P* < 0.001), urinary protein level (WMD = 0.76, *P* = 0.001), urinary protein ≥ 1.5 g/d (OR = 1.98, *P* < 0.001), lambda − chain value (WMD = 29.02, *P* < 0.001), abnormal free light-chain ratio (OR = 4.16, *P* = 0.002), bone marrow puncture rate (OR = 5.11, *P* = 0.019), and abnormal bone marrow outcome rate (OR = 9.63, *P* = 0.005). No statistical significance was found for the remaining clinical features: serum creatinine (WMD = 0.08, *P* = 0.293), estimated glomerular filtration rate (WMD = − 5.63, *P* = 0.056), sex (OR = 0.81, *P* = 0.449), hemoglobin (WMD = − 0.26, *P* = 0.554), complement C3 (WMD = − 14.43, *P* = 0.189), low complement C3 ratio (OR = 2.93, *P* = 0.222), complement C4 (WMD = − 2.68, *P* = 0.293), hematuria (OR = 0.86, *P* = 0.733), and kappa − chain value (WMD = 23.84, *P* = 0.509). The results are shown in Table 2. We also calculated pooled ORs for five potentially independent risk factors (urine protein ≥ 1.5 g/d, diabetes, sex, hematuria, and abnormal free light-chain ratio) by multivariable logistic regression analysis, involving two studies (Table 3). All indicators included in the analyses were screened in the original articles by univariable logistic regression combined with clinical experience, and the number of variables was restricted to 10 events per one predicting variable.

**Table 2:** Meta-analysis of clinical features of MGRS

Clinical Features	Studies	Effects model	OR/WMD	95%CI	P-value	Heterogeneity	
						I^2^	P-value
Age(year)	4	Fixed	1.78	0.21–3.35	0.026	1.8	0.383
Sex(%)	4	Random	0.81	0.47–1.40	0.449	56.6	0.075
Hypertension(%)	2	Fixed	0.54	0.4–0.73	<0.001	0.0	0.571
Diabetes(%)	3	Fixed	0.42	0.29–0.59	<0.001	0.0	0.669
Albumin(g/dl)	3	Fixed	-0.26	-0.38–0.14	<0.001	0.0	0.450
Hemoglobin(g/dl)	3	Random	-0.26	-1.12-0.60	0.554	72.4	0.027
Serum creatinine(mg/dl)	4	Fixed	0.08	-0.07-0.22	0.293	0.0	0.558
eGFR(ml/min/1.73 m2)	3	Fixed	-5.63	-11.41-0.16	0.056	0.0	0.869
complement C3(mg/dl)	2	Random	-14.43	-35.96-7.10	0.189	92.6	<0.001
low complement C3 ratio(%)	2	Random	2.93	0.52–16.46	0.222	64.9	0.091
complement C4(mg/dl)	2	Random	-2.68	-7.68-2.32	0.293	73.0	0.054
urinary protein level(g/d)	4	Fixed	0.76	0.31–1.20	0.001	42.0	0.160
urinary protein ≥ 1.5 g/d(%)	3	Fixed	1.98	1.46–2.68	<0.001	7.0	0.341
hematuria(%)	4	Random	0.86	0.36–2.06	0.733	84.8	<0.001
k-chain value(mg/l)	2	Random	23.84	-46.98-94.67	0.509	96.0	<0.001
λ-chain value(mg/l)	2	Fixed	29.02	16.55–41.49	<0.001	0.0	0.593
abnormal free light chain ratio(%)	3	Random	4.16	1.65–10.47	0.002	77.1	0.013
bone marrow puncture rate(%)	3	Random	5.11	1.31–19.95	0.019	89.8	<0.001
abnormal bone marrow outcome rate(%)	2	Random	9.63	1.98–46.88	0.005	90.5	0.001

**Table 3:** Meta-analysis of independent risk factors for MGRS

Independent risk factors	Effects model	OR	95%CI	P-value	Heterogeneity	
					I^2^	P-value
urinary protein ≥ 1.5 g/d(%)	Fixed	2.80	1.53–5.15	0.001	0.0	0.528
abnormal free light chain ratio(%)	Fixed	6.98	4.10-11.91	<0.001	28.4	0.237
sex(%)	Random	1.04	0.27–4.01	0.958	81.2	0.021
hematuria(%)	Random	1.71	0.65–4.52	0.275	68.7	0.074
diabetes(%)	Fixed	0.58	0.31–1.07	0.081	0.0	0.687

#### Meta-analysis of the clinical features of amyloidosis

Given that amyloidosis is the most prevalent type of MGRS, we also compared MGRS-associated amyloidosis with other MGRS types. We evaluated pooled effect sizes for 23 clinical characteristics (age, sex, diabetes, stage CKD3, stage CKD4, stage CKD5, albumin, hemoglobin, serum creatinine, estimated glomerular filtration rate, complement C3, complement C4, urinary protein level, urinary protein ≥ 1.5 g/d, nephrotic range proteinuria, hematuria, monoclonal, biclonal, kappa-chain value, lambda-chain value, abnormal free light-chain ratio, bone marrow puncture rate, and abnormal bone marrow outcome rate). The *P* value was statistically significant among the following 14 clinical features: stage CKD3 (OR = 0.26, *P* < 0.001), stage CKD4 (OR = 0.44, *P* = 0.003), stage CKD5 (OR = 0.27, *P* < 0.001), estimated glomerular filtration rate (WMD = 34.24, *P* < 0.001), complement C3 (WMD = 30.78, *P* < 0.001), urinary protein level (WMD = 2.12, *P* < 0.001), urinary protein ≥ 1.5 g/d (OR = 7.08, *P* < 0.001), nephrotic range proteinuria (OR = 3.65, *P* < 0.001), abnormal free light-chain ratio (OR = 3.71, *P* < 0.001), abnormal bone marrow outcome rate (OR = 4.30, *P* = 0.001), age (WMD = 3.52, *P* = 0.006), albumin (WMD = − 0.55, *P* < 0.001), serum creatinine (WMD = − 0.66, *P* = 0.023), and hematuria (OR = 0.31, *P* = 0.023). No statistically significant differences were found for the other clinical features: sex (OR = 1.14, *P* = 0.389), monoclonal (OR = 1.33, *P* = 0.569), biclonal (OR = 0.75, *P* = 0.569), bone marrow puncture rate (OR = 1.09, *P* = 0.806), diabetes (OR = 0.78, *P* = 0.803), hemoglobin (WMD = 1.70, *P* = 0.094), complement C4 (WMD = 2.48, *P* = 0.667), kappa − chain value (WMD = − 8.99, *P* = 0.202), and lambda − chain value (WMD = 8.72, *P* = 0.159). All results are shown in Table 4.

**Table 4:** Meta-analysis of clinical features of amyloidosis

Clinical Features	Studies	Effects model	OR/WMD	95%CI	P-value	Heterogeneity	
						I^2^	P-value
Age(year)	4	Random	3.52	1.00-6.05	0.006	51.0	0.106
Sex(%)	4	Fixed	1.14	0.84–1.55	0.389	0.0	0.783
diabetes(%)	2	Random	0.78	0.11–5.46	0.803	81.5	0.020
stage CKD3(%)	2	Fixed	0.26	0.16–0.42	<0.001	10.6	0.290
stage CKD4(%)	2	Fixed	0.44	0.26–0.76	0.003	0.0	0.976
stage CKD5(%)	2	Fixed	0.27	0.13–0.55	<0.001	0.0	0.431
Albumin(g/dl)	2	Random	-0.55	-0.82–0.28	<0.001	63.0	0.100
Hemoglobin(g/dl)	2	Random	1.70	-0.29-3.70	0.094	87.4	0.005
serum creatinine(mg/dl)	3	Random	-0.66	-1.22–0.09	0.023	68.8	0.040
eGFR(ml/min/1.73 m2)	2	Fixed	34.24	27.88–40.59	<0.001	0.0	0.443
complement C3(mg/dl)	2	Fixed	30.78	23.34–38.22	<0.001	0.0	0.635
complement C4(mg/dl)	2	Random	2.48	-8.80-13.76	0.667	86.9	0.006
urinary protein level(g/d)	3	Fixed	2.12	1.46–2.79	<0.001	0.0	0.928
urinary protein ≥ 1.5 g/d(%)	2	Fixed	7.08	3.80-13.18	<0.001	0.0	0.609
nephrotic range proteinuria(%)	2	Fixed	3.65	2.28–5.85	<0.001	0.0	0.335
hematuria(%)	2	Random	0.31	0.11–0.85	0.023	69.5	0.070
monoclonal(%)	2	Fixed	1.33	0.49–3.60	0.569	0.0	0.877
bi-clonal(%)	2	Fixed	0.75	0.28–2.02	0.569	0.0	0.877
k-chain value(mg/l)	2	Random	-8.99	-22.81-4.83	0.202	81.8	0.019
λ-chain value(mg/l)	2	Random	8.72	-3.42-20.87	0.159	85.1	0.010
abnormal free light chain ratio(%)	2	Fixed	3.71	1.82–7.56	<0.001	0.0	0.405
bone marrow puncture rate(%)	2	Fixed	1.09	0.53–2.24	0.806	12.9	0.284
abnormal bone marrow outcome rate(%)	2	Fixed	4.30	1.80-10.28	0.001	0.0	0.910

In the meta-analysis of some clinical indicators, heterogeneity was high. Because the number of studies in each indicator was so minimal, sensitivity analyses and meta-regressions were not performed to investigate the causes of heterogeneity.

## Discussion

One previous estimate of the prevalence of chronic kidney disease (CKD) in China was 10.8% [16]. A recent study showed that a higher prevalence of CKD was observed in elderly people [17]. The estimated prevalence of MGUS was 3.2% in individuals > 50 years and 5.3% in those > 70 years [18]. Patients with MIg and concomitant kidney diseases are frequently found in clinical practice. MGRS accounted for 2–10% of the MGUS population according to previous studies [19–21]. Our research showed that MGRS was responsible for 41% of MG patients who had renal disease and received renal biopsy. The prevalence was similar in studies from China and America. According to the IKMG consensus, kidney biopsy is the only way to diagnose MGRS, and studies have shown that patients with MGRS are not at high risk of bleeding after a kidney biopsy [3, 22]. For older patients, a kidney biopsy is still recommended because epidemiological studies have shown that the incidence of MGRS increases with age [8]. However, the indications for renal biopsy may vary among different medical units. Biopsy might not be recommended for elderly patients with a risk of bleeding during renal biopsy. Even so, physicians should be aware that MGRS is still a significant and frequent cause of kidney damage in patients with MIg and renal disease.

Amyloidosis is the leading type of MGRS followed by MIDD, cryoglobulinemia, and proliferative glomerulonephritis with monoclonal immunoglobulin deposits (PGNMID), with prevalences of 11%, 6%, and 6%, respectively. The prevalences of other types were ≤ 5%. PGNMID is characterized predominantly by membranoproliferative glomerulonephritis [23]. Immunofluorescence and electron microscopic studies show granular deposits involving glomeruli only and composed of monotypic immunoglobulin G (IgG, mainly IgG3-κ). PGNMID do not have clinical or laboratory evidence of cryoglobulinemia. Recently, heavy-chain/light-chain (HLC) antibodies targeting conformational epitopes at the junctions of the HLC regions (CH1 and CL) are introduced to quantify intact HLC pairs. Compared with other current techniques, HLC immunofluorescence has higher specificity for detection of intact MIg, which excluded 31% PGNMID [24]. Therefore, the prevalence of PGNMID may decrease with application of HLC antibodies.

In this study, patients with MGRS were older and had fewer underlying diseases (diabetes and hypertension), lower serum albumin, more urinary protein (including proteinuria ≥ 1.5 g/d ratio), more abnormal free light-chain ratios, and more abnormal findings from the bone marrow than those of patients with MG and non-MIg-associated nephropathy [8, 9, 13, 14]. Further analysis revealed that patients with amyloidosis were older and had higher levels of eGFR, higher serum C3 values, more urinary protein, more abnormal free light-chain ratios, more abnormal bone marrow outcomes, lower serum albumin and creatinine, and fewer cases of hematuria than those with non-amyloidosis MGRS [8, 9, 12, 15]. Amyloidosis is the leading type of MGRS, with a prevalence of 64%. Amyloidosis is mainly characterized by proteinuria, even in the nephrotic-syndrome range, rare hematuria, hypoalbuminemia, and fairly normal eGFR. It is not surprising that the clinical features of MGRS are similar to those of amyloidosis. It is worth noting that the heterogeneity of some of the indicators in the above studies and the inability to further investigate the source of the heterogeneity weakened the reliability of the findings for the diagnosis of MGRS.

Two studies have assessed independent indicators for the diagnosis of MGRS [8, 9]. Pooled analysis revealed that an abnormal free light-chain ratio and urine protein ≥ 1.5 g/d were independent risk factors for the diagnosis of MGRS. Since amyloidosis is the leading subtype in MGRS proteinuria is the main clinical presentation of if, in this meta-analysis, urine protein ≥ 1.5 g/d may reflect the great proportion of amyloidosis in MGRS. It should be noted that urinary protein < 1.5 g/d can also be observed in MGRS-associated lesions with non-glomerular involvement, including light-chain proximal tubulopathy, and amyloidosis with primarily interstitial and vascular damage, etc. It is crucial to search non-amyloidosis MGRS in these patients although they only contribute to a small part of MGRS. In most cases, a free light-chain ratio outside the range of 0.27–1.65 in patients with eGFR ≥ 60 ml/min/1.73 m^2^ or outside the range of 0.37–3.10 in patients with eGFR < 60 ml/min/1.73 m^2^ indicates an abnormal free light-chain ratio. An abnormal free light-chain ratio reflects a greater MIg burden. Although MGRS mainly injures kidneys in ways other than those related to a tumor burden, a greater MIg burden may aggravate MGRS and become a clinical indicator for diagnosis of MGRS. At first, lamda chain value was shown to be significant in MGRS. However, multivariable logistic regression analysis show that only abnormal free light chain ratio and urine protein over 1.5 g/24 h predict MGRS. Monoclonal lamda light chain is dominant in MIg-related amyloidosis which is the leading subtype of MGRS. Abnormal lamda chain value may mainly reflect clinical profile of amyloidosis. However, kappa chain may be dominant in non-amyloidosis MGRS, such as MIDD. Abnormal free light chain ratio may reflect overall profile of MGRS. Recently, the difference between involved and uninvolved free light chain (dFLC) is used to assess treatment response for amyloidosis. Serum dFLCs reflect the burden of MIg, which are not affected by renal function [25]. The quantitative mass spectrometry is a more sensitive approach to measure MIg, enabling detection of very low disease burden, including minimal residual disease following therapy [26]. It remains unknown if the very low MIg measured by the quantitative mass spectrometry independently predicts the diagnosis of MGRS.

The pooled analysis showed that neither the univariate model nor multivariate model showed that hematuria was a statistically significant predictor of MGRS. A Mayo Clinic study suggested that proteinuria ≥ 1.5 g/d, hematuria and an elevated free light-chain ratio increased the likelihood of finding MGRS [8]. However, Zhao MH’s study from China only revealed the effect of proteinuria ≥ 1.5 g/d and an elevated free light-chain ratio. The most typical symptom of IgA nephropathy, which is the most prevalent glomerular disease in China, is hematuria [27]. Therefore, in Chinese people with both renal impairment and MG, hematuria may not be a reliable predictor of MGRS. It is still necessary to thoroughly evaluate valid results by merging research from different geographic and racial groupings, nevertheless.

## Limitations

To our knowledge, no meta-analysis related to the clinical characterization of MGRS has been published previously. Our study had the following limitations. First, due to the reference range of the indicators covered, ethnicity, regional disparities, and retrospective research design, high variability in the results for some indicators was expected, which skewed the results. Although the analysis used a random-effects model, the study’s heterogeneity was maintained. Due to the limited number of studies, we could not conduct further subgroup analysis or meta-regression analysis, making difficult to understand where the heterogeneity came from. In fact, MGRS itself has significant heterogeneity. MGRS contains many kinds of subtypes, such as amyloidosis, MIDD, et al. These subtypes present with varying clinicopathological features. The broad spectrum of MGRS in studies may contribute to the heterogeneity of this meta-analysis partially. Otherwise, the results of the meta-analyse may be influenced by the leading subtype of MGRS. In this study, the clinical features of MGRS may reflect MGRS-related amyloidosis since the latter accounts for the largest proportion of MGRS. However, due to the lack of primary data, our study was only a meta-analysis of secondary data and we were unable to re-group to analyze the diagnostic characteristic factors of amyloidosis for the time being. Second, the majority of clinical factors were not examined using multivariate modeling, which might have resulted in inaccurate findings. Third, clinical indicators of organ involvement other than of the kidney, such as NT-proBNP for amyloid-related cardiac lesions, were lacking in the included studies, and should be included in future relevant studies. Finally, the overall number of studies was low, and the number of studies for each indicator varied, which affects how broadly the conclusions can be applied.

## Conclusion

We found that MGRS accounted for 41% of patients with MG who had kidney disease and had undergone renal biopsy. MIg-related amyloidosis is the leading type of MGRS. Proteinuria ≥ 1.5 g/d and an abnormal free light-chain ratio were independent risk factors for the diagnosis of MGRS.

## Data Availability

All data generated or analyzed during this study are included in this article. Further enquiries can be directed to the corresponding author.

## Abbreviations

MGRS: Monoclonal gammopathy of renal significance
MGs: Monoclonal gammopathies
MIg: Monoclonal immunoglobulin
MGCS: Monoclonal gammopathy of clinical significance
IKMG: International Kidney and Monoclonal Gammopathy Research Group
MGUS: Monoclonal gammopathy of undetermined significance
NOS: Newcastle–Ottawa Scale
WMD: Weighted mean difference
OR: Odds ratio
CKD: Chronic kidney disease
IgG: Monotypic immunoglobulin G
HLC: Heavy-chain/light-chain
dFLC: The difference between involved and uninvolved free light chain
MIDD: Monoclonal immunoglobulin deposition disease
PGNMID: Proliferative glomerulonephritis with monoclonal immunoglobulin deposits
